# Knowledge, Attitudes, and Practices of Dietary Supplement Use in Western Saudi Arabia: A Cross-Sectional Study

**DOI:** 10.3390/nu17071233

**Published:** 2025-04-01

**Authors:** Abdullah A. Alshehri, Sara Alqahtani, Reuof Aldajani, Batool Alsharabi, Waad Alzahrani, Ghadi Alguthami, Wael Y. Khawagi, Hassan Arida

**Affiliations:** 1 Department of Clinical Pharmacy, College of Pharmacy, Taif University, Taif 21944, Saudi Arabia; w.khawagi@tu.edu.sa; 2 College of Pharmacy, Taif University, Taif 21944, Saudi Arabia; 3 Department of Pharmaceutical Chemistry, College of Pharmacy, Taif University, Taif 21944, Saudi Arabia; aridaha@hotmail.com

**Keywords:** dietary supplements, attitude, knowledge, practice, Saudi Arabia

## Abstract

Background: Dietary supplements (DSs) are widely used to complement diets, particularly among individuals with specific nutritional needs. While DSs can promote health and prevent disease, improper use may lead to adverse effects or medication interactions, highlighting the need for safe, informed consumption. This study assesses knowledge, attitudes, and practices (KAP) related to DSs in Western Saudi Arabia, providing insights into national consumption patterns. Method: A cross-sectional online survey was conducted between December 2023 and February 2024. A 28-item questionnaire covering demographics, knowledge, attitudes, and practices regarding DSs was administered. Participants aged 18 years and older residing in the study regions were recruited through convenience sampling. Data were analyzed with SPSS version 22 using descriptive statistics and chi-square tests. Results: A total of 1006 participants completed the survey, with 70.5% (*n* = 709) reporting DS use. Most respondents were female (71.8%) and 34.3% were aged between 18 and 30 years. Although 82.5% were aware of DSs, only 41% knew the correct dosages, and 30.3% expressed uncertainty about DS safety. About 28.1% of DS users experienced side effects. Multivitamins were the most commonly used DS type (44.9%), followed by mono-vitamins (39.9%) and minerals (7.5%). The primary motivations for DS use were health improvement and ensuring adequate nutrition (66%), while 20% used DSs to address specific deficiencies. Most participants sourced DSs from pharmacies (85.5%), and 46.6% relied on the internet for information. Significant differences in DSs use were observed based on gender and age (*p* < 0.001). Conclusion: This study highlights the widespread use of DSs, with significant gaps in knowledge, attitudes, and practices concerning dosage and safety. Associations between DS use and demographic factors indicate that public health strategies should address these variables. Targeted education and clear guidance on safe DS use are essential for promoting informed consumption and reducing potential health risks.

## 1. Introduction

A balanced diet is essential for promoting health, preventing disease, and supporting bodily functions, including muscle performance, immune strength, and stress management [1,2]. Although a well-balanced diet provides essential nutrients, dietary supplements (DSs) have gained popularity as a complement to dietary intake [3]. The U.S. Food and Drug Administration (FDA) defines DSs as products containing dietary ingredients, such as vitamins, minerals, herbs, and amino acids, intended to supplement the diet [4]. According to the Dietary Supplement Health and Education Act (DSHEA) of 1994, DSs are designed for oral consumption and serve as supplemental sources of essential nutrients [5]. DSs may consist of individual nutrients or combinations, such as multivitamins, amino acids, botanicals, and minerals such as magnesium and zinc [1]. They are available in various forms, including tablets, capsules, liquids, and powders [6]. Increased health awareness, education, and DSs accessibility have driven global consumption. In Saudi Arabia, for instance, the DSs market was valued at USD 294.7 million in 2021, with projections to reach USD 605.83 million by 2028, indicating substantial growth in consumer demand [7,8]. However, this accounts for only a small share of the global dietary supplement industry, which was valued at USD 177.5 billion in 2023. Despite economic growth, malnutrition remains a concern in Saudi Arabia, with iron, vitamin D, and other micronutrient deficiencies particularly affecting children and pregnant women [9,10].

Motivations for DS use vary widely, with individuals turning to DSs to support health, ensure adequate nutrition, enhance physical appearance, or aid weight management [11]. For example, vitamin C is popular for boosting immunity, while vitamin D and calcium are commonly taken to support bone health, particularly among older people [12,13]. Certain groups with unique nutritional needs, like pregnant women, athletes, and individuals recovering from illness, may particularly benefit from DSs [14,15]. Moreover, nutritional deficiencies can lead to serious health issues such as chronic metabolic disorders, marasmus, and kwashiorkor [16]. However, research indicates that DSs are often consumed by individuals who are already well-nourished rather than those at risk of deficiencies. Higher-income individuals with balanced diets are more likely to use supplements, whereas vulnerable groups, such as the elderly and low-income populations, may have limited access or awareness [17]. Despite their benefits, excessive DSs consumption can lead to adverse effects. Certain supplements have been associated with liver and renal toxicity, suppressed immune function, birth defects, and gastrointestinal disturbance [18,19]. Additionally, DSs can interact with medications, potentially reducing their effectiveness. St. John’s wort, for instance, may interfere with antidepressants and birth control pills [20]. Thus, understanding safe DSs use is critical, particularly as some individuals may rely on supplements instead of nutrient-rich foods [21]. Educational programs and access to credible information sources, including healthcare professionals, are essential to promote the responsible DSs usage [1,22].

Given the growing prevalence of DSs use, understanding the public knowledge, attitudes, and practices (KAP) regarding DSs is essential to promote safe and informed usage [19]. Numerous studies have investigated DSs consumption patterns, highlighting significant knowledge gaps and unsafe practices in Jordan, the UAE, Bahrain, and Lebanon [1,2,23,24,25]. While global dietary supplement consumption trends are well documented, data from Saudi Arabia remain limited, particularly in regions with diverse urban and rural populations. To date, only two studies have explored KAP [3,12]. One focused on specific age groups within a particular region, and the other on university students. Therefore, this study aims to provide a comprehensive understanding of DSs knowledge, attitudes, and practices in Western Saudi Arabia, including the populous Mecca and Medina provinces and addressing both urban and rural populations. This research offers insights into public DSs use and contributes to a broader understanding of DSs consumption across Saudi Arabia.

## 2. Method

### 2.1. Study Design and Setting

A cross-sectional survey was conducted among the general population, including citizens and residents from both urban and rural areas in the Western regions of Saudi Arabia, between December 2023 and February 2024.

### 2.2. Questionnaire Development

A self-administered, 28-item questionnaire was developed based on prior studies and refined through consultation with an academic expert pharmacist [1,2,6,12]. The questionnaire included an introductory section that outlined the study’s objectives, informed participants of the voluntary nature of their participation, and sought their consent before beginning the survey.

The questionnaire was divided into four sections. Section 1 gathered demographic information through ten questions, covering participants’ age, gender, marital status, city of residence, education level, employment status, monthly income, chronic disease status, lifestyle factors such as smoking and diet, and DS usage. Section 2 focused on knowledge, with five questions assessing participants’ understanding of DSs, including their knowledge of benefits, risks, and recommended usage guidelines. Section 3 addressed attitudes through three questions, evaluating participants’ perceptions of DSs, including their views on effectiveness, safety, and necessity for health and well-being. Section 4 examined participants’ practices related to DS usage. using ten questions, exploring participants’ DS usage patterns, such as frequency, types of supplements used, and reasons for consumption. The questionnaire, distributed in Arabic, was expected to take approximately seven minutes to complete. The questionnaire was pilot-tested with a sample of 10 participants from the target population to assess clarity, relevance, and comprehensibility. Feedback from the pilot study was used to refine wording and ensure alignment with the study’s objectives. Data from the pilot study were not included in the analysis.

### 2.3. Participant Recruitment and Data Collection

Participants were recruited via convenience sampling on online platforms, capitalizing on the high internet usage in Saudi Arabia. Data were collected through a self-administered, pretested Google Forms survey link distributed via social media platforms like WhatsApp and Telegram groups. Eligibility criteria included participants being 18 years or older, residing in the Western regions of Saudi Arabia, and voluntarily agreeing to participate. Individuals under 18 or living outside the Western regions were excluded.

### 2.4. Sample Size Calculation

The sample size was calculated using Cochran’s equations for an unlimited population:

n = z^2^ × p (1 − p)/e^2^

where n represents the sample size, z is the z-score for a 95% confidence level (1.96), p is the sample proportion (0.50), and e is the margin of error (0.05). Based on these values, the required sample size was calculated as 384 participants.

### 2.5. Statistical Analysis

Data were analyzed using the Statistical Package for Social Sciences (SPSS), version 22 (IBM Corp., Armonk, NY, USA). Descriptive statistics, including frequencies and percentages, were used to summarize participants’ responses. The chi-square test was applied to examine associations between categorical variables, with statistical significance set at a *p*-value of <0.05.

### 2.6. Ethical Approval

The study obtained ethical approval from the Taif University Ethical Committee (Approval No: HAO-02-T-105; Application No: 45-066) on 13 November 2023. Participants provided informed consent before beginning the survey, in accordance with ethical guidelines. Their personal information was kept confidential and anonymous throughout the study. To ensure data confidentiality, no personal identifiers were collected.

## 3. Results

### 3.1. Demographic Characteristics of Participants (n = 1006)

A total of 1006 participants completed the questionnaire, although the response rate could not be calculated. Most respondents were female (71.8%, *n* = 722), and 34.3% (*n* = 345) were aged between 18 and 30 years. Nearly half of the participants resided in Taif (46%, *n* = 463), and 44.3% (*n* = 446) were employed in the government sector. Most participants (74.9%, *n* = 753) held a university degree, and 63.3% (*n* = 637) were married. In terms of income, 38.3% (*n* = 385) reported earning more than 10,000 SR per month. The majority of participants (74.8%, *n* = 752) had no chronic illnesses. Regarding lifestyle factors, 17.1% (*n* = 172) reported as athletes, and 10.2% (*n* = 103) followed a specific diet (see Table 1).

A total of 709 participants (70.5%) were identified as dietary supplement (DS) users, while 297 participants (29.5%) were classified as non-users. Significant demographic differences were observed between DS users and non-users. Among males, 54.2% reported DS use, while 45.8% did not. Among females, 76.9% were DS users compared to 23.1% who were non-users (*p* < 0.001). DS use was most prevalent among those aged 18–30 (74.5%), followed by those aged 31–40 (80.2%). Non-use was highest among participants aged 50 or older (40.8%) (*p* < 0.001). The analysis also revealed statistically significant differences in DS usage based on city of residence (*p* = 0.023) and employment status (*p* < 0.001). However, no statistically significant differences were found regarding educational level, marital status, chronic disease status, income, or lifestyle factors (see Table 1).

### 3.2. Participants’ Knowledge of Dietary Supplements

**Figure 1:**
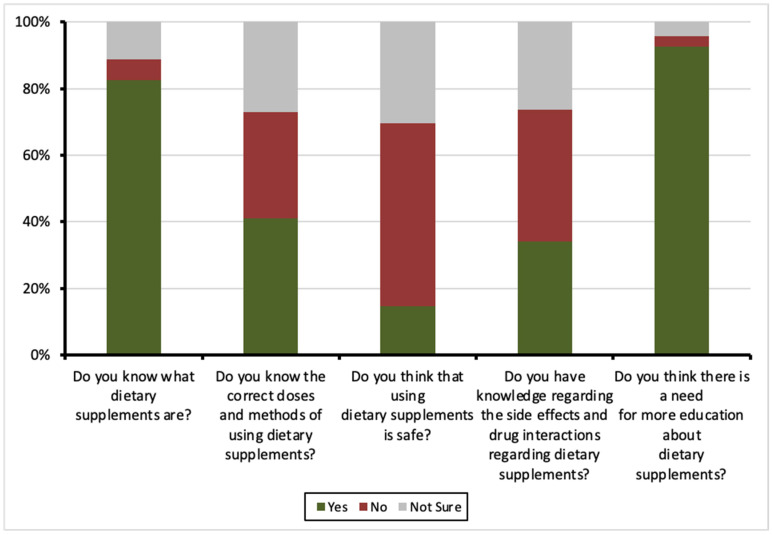
Knowledge assessment of dietary supplements among participants.

Most respondents (82.5%, *n* = 830) were aware of what DSs are, but only 41% (*n* = 412) knew the correct doses and methods of use. A notable portion of participants (27.1%, *n* = 273) expressed uncertainty regarding proper usage. When asked about the safety of DSs, 55% (*n* = 553) believed they were not safe, while 30.3% (*n* = 305) were uncertain. Knowledge of potential side effects and drug interactions was limited, with 39.7% (*n* = 399) of respondents unaware of them. However, an overwhelming majority (92.6%, *n* = 932) acknowledged the need for more education on DSs, highlighting a clear gap in understanding. Figure 1 shows the distribution of responses to knowledge-related questions about DSs.

### 3.3. Participants’ Attitudes Toward Dietary Supplements

**Figure 2:**
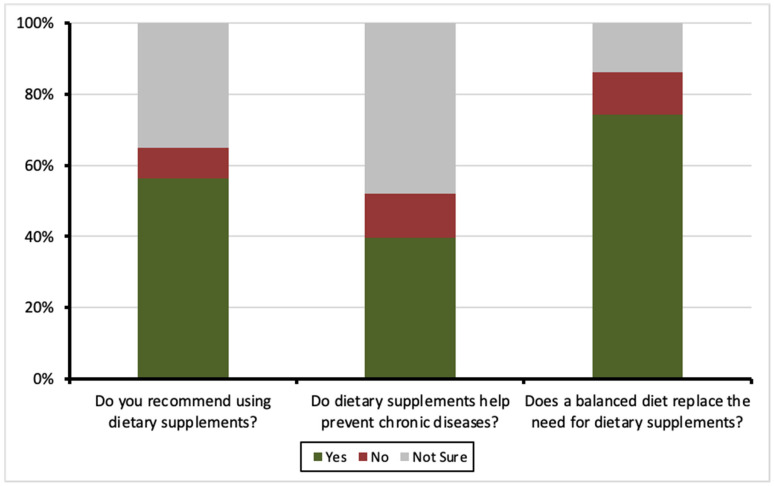
Attitudes toward dietary supplements among participants.

More than half of the respondents (56.3%, *n* = 566) recommended the use of DSs to others. When asked about the role of DSs in preventing chronic conditions, 39.6% (*n* = 398) expressed confidence in their potential benefits, while 47.9% (*n* = 482) were uncertain. Furthermore, a majority (74.4%, *n* = 748) agreed that a nutritious and balanced diet can adequately replace the need for DSs. Figure 2 demonstrates the responses to attitude-related questions about DSs.

### 3.4. Participants’ Practices Regarding Dietary Supplements (n = 709)

**Figure 3:**
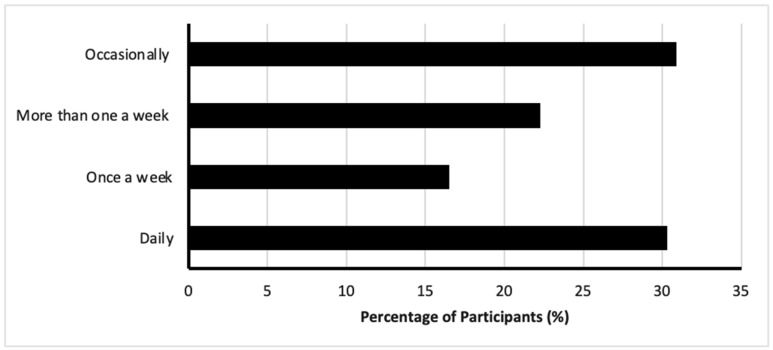
Frequency of dietary supplement usage among participants.

**Figure 4:**
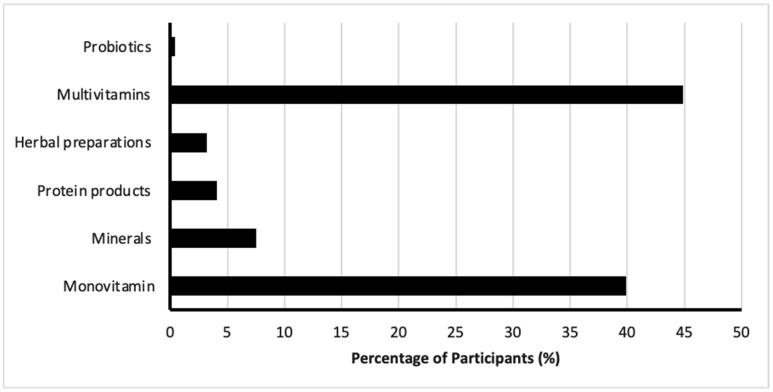
Types of dietary supplements most commonly used by participants.

Among the DS user, participants reported various practices in DS usage. Approximately a third of participants (30.3%, *n* = 215) indicated using DSs daily, while 30.9% (*n* = 219) reported using them occasionally (Figure 3). The most commonly used type of DSs was multivitamins, reported by 44.9% (*n* = 377), followed by mono-vitamins (39.9%, *n* = 299), minerals (7.5%, *n* = 56), protein products (4.1%, *n* = 31), natural herbal preparations (3.2%, *n* = 24), and probiotics (0.4%, *n* = 3) (Figure 4).

**Figure 5:**
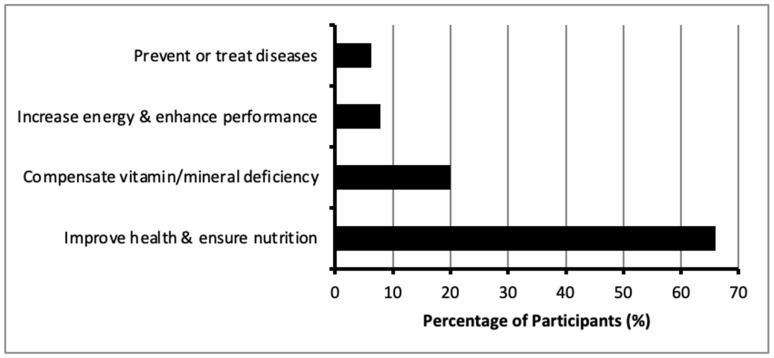
Reasons for dietary supplement consumption among participants.

Tablets were the most common form of DSs used, with 68.4% (*n* = 518) of participants opting for them, followed by capsules at 26.7% (*n* = 202). Most participants obtained their supplements from pharmacies (85.5%, *n* = 647) and nutrition stores (4.9%, *n* = 37). Information about DSs was primarily sourced from the internet (46.6%, *n* = 367) and healthcare providers (42.6%, *n* = 335). Particularly, 68.8% (*n* = 488) of participants expressed consulting a doctor before using supplements, while 31.2% (*n* = 221) did not seek medical advice prior to DS use. Regarding side effects, 71.9% (*n* = 510) of DS users reported no adverse effects, whereas 28.1% (*n* = 199) experienced some side effects. The main reasons for DS use were to improve health and ensure adequate nutrition (66%, *n* = 664), with others citing compensation for vitamin or mineral deficiencies (20%, *n* = 201). Fewer participants used DSs to increase energy (7.8%, *n* = 78) or for disease prevention and treatment (6.3%, *n* = 63) (Figure 5).

## 4. Discussion

This study highlights a high prevalence of DS use, particularly multivitamins, with significant variations observed across gender, age, city of residence, and employment status. Younger individuals were more likely to use DSs, with females also constituting a majority of users, indicating distinct demographic trends in DS consumption. While a large proportion of participants reported being aware of DSs, many lacked detailed knowledge about correct dosages and proper usage, and over half expressed uncertainty regarding DS safety. Although the majority of DS users reported no adverse effects, some did experience side effects. Additionally, most participants expressed a need for further education on DSs, underscoring a gap in understanding potential risks and benefits. The main motivations for DS use included improving health, ensuring adequate nutrition, and addressing specific nutrient deficiencies. These findings emphasize the importance of targeted educational initiatives to bridge knowledge gaps and promote the safe consumption of DSs.

The prevalence of DSs usage observed in this study (70.5%) aligns with findings from similar research. In Jordan, 60.9% of participants reported DSs use [1], and in other studies from Saudi Arabia, usage rates of 55.3% and 50.7% were reported [3,11]. Our findings also show that more than three-quarters (78.3%) of DS users were female, a trend echoed in several studies. This may reflect unique nutritional needs among women, particularly during life stages such as pregnancy and post-menopause, as well as evidence suggesting that women prioritize health more than men [24,26]. These findings are consistent with a study in Jordan, which reported that 47.6% of DS users were female [25], and another in Saudi Arabia, where the rate was 86% [11]. However, in contrast to our results, a study in the Asir region of Saudi Arabia reported higher DS usage among males [3]. Furthermore, 94% of DSs users in our study were non-smokers, reflecting a generally health-conscious lifestyle. This is consistent with findings in Saudi Arabia, where one study in the Asir region reported a non-smoking rate of 80.2% among middle-aged DS users and 98.9% among elderly users, while another study found a 94.3% non-smoking rate among DS users [3,11]. This pattern suggests that DSs users tend to adopt other healthy habits, such as regular exercise and mindful eating [11].

Motivations for DS use were also consistent with previous studies. In this study, 66% of participants used DSs for health improvement and nutritional adequacy, similar to findings from Saudi Arabia, the UAE, and Jordan, where primary motivations included maintaining health, ensuring nutrition, and boosting energy levels [1,3,25]. Regarding information sources on DSs, our findings indicate that the internet and social networks were primary sources, followed by nutrition and health specialists. This finding aligns with other studies identifying online platforms as the main information source for DSs [1,11]. However, while convenient, the internet does not always provide reliable information, which can lead to misunderstandings and DS misuse [1,11]. Studies from Jordan and the UAE showed different trends, with participants relying more heavily on healthcare professionals for DS information [2,25]. Multivitamins were the most commonly used types of DSs in our study, similar to findings from studies in Saudi Arabia [3,11]. However, a study in Jordan indicated a preference for single vitamins or minerals over multivitamins, suggesting regional variation in DS preferences [1].

This study provides a clear message for healthcare professionals, policymakers, and public health educators about the widespread use of DSs in Saudi Arabia. It emphasizes the need for improved education, regulation, and targeted public health interventions to ensure safe and informed consumption. Addressing knowledge gaps and promoting healthcare consultations before DS use are crucial to mitigating potential risks. Tailored awareness campaigns, clear labeling, and healthcare guidance are essential for responsible DS consumption [27]. To prevent overuse, individuals should monitor intake, recognize overdose symptoms, undergo regular health check-ups, and consider drug–supplement interactions [28]. DS use should be guided by medical advice rather than trends, with any sudden health changes prompting a reassessment of intake [29].

Future research should include detailed comparative analyses of knowledge, attitudes, and practices between DS users and non-users. Qualitative research may offer deeper insights into motivations and perceptions driving DS use, particularly among high-consumption groups. Additionally, evaluating the effectiveness of educational interventions led by pharmacists and other healthcare providers, as well as assessing the prevalence of DS–medication interactions, would provide valuable guidance for targeted public health strategies.

This study provides the first comprehensive assessment of DSs KAP across urban and rural areas in various provinces of Saudi Arabia, based on a large and diverse sample. By focusing on demographics, the study offers valuable insights for tailoring public health interventions. The use of a validated Arabic questionnaire further enhances the reliability and cultural relevance of the findings. However, several limitations must be acknowledged. The convenience sampling method may limit the generalizability of the findings, and reliance on self-reported data introduces potential response bias. Additionally, recruitment through social media platforms may have led to selection bias, underrepresenting individuals with lower socioeconomic status, limited digital access, or lower education levels. Furthermore, while social media platforms provide general user metrics, the exact number of individuals who viewed the survey remains unknown. Consequently, our sample represents a small fraction of the broader population and may not be fully generalizable. These populations may have distinct DS usage patterns and a higher risk of improper use, further affecting the study’s applicability. Unmeasured factors, such as supplement affordability, perceived health changes, and specific health concerns, may have also influenced DS consumption. Notably, most participants reported being in good health without chronic illnesses, which could have biased the results by underrepresenting individuals with medical conditions who may use DSs differently. Moreover, volunteer bias may have impacted the sample’s representativeness, as individuals with positive attitudes toward DSs were more likely to participate. Furthermore, the response rate could not be determined due to the open nature of social media recruitment.

## 5. Conclusions

This study highlights the widespread use of DSs to improve health and ensure adequate nutrition. Significant gaps in KAP regarding correct dosages and safety indicate a need for targeted educational efforts. Associations between DS use and demographic factors, such as gender, age, and location, suggest that public health initiatives should consider these variables to effectively reach diverse groups. Promoting awareness and providing guidance on safe DS use are essential for fostering informed consumption, reducing potential risks, and improving public health outcomes.

## Figures and Tables

**Table 1:** Social, demographic, and economic characteristics of the study sample (*n* = 1006).

Characteristic		Total *n* (%)	User of DSs *n* (%)	Non-User of DSs *n* (%)	*p*-Value
Gender					<0.001
	Male	284 (28.2)	154 (54.2)	130 (45.8)	
	Female	722 (71.8)	555 (76.9)	167 (23.1)	
Age					<0.001
	18–30	345 (34.3)	257 (74.5)	88 (25.5)	
	31–40	172 (17.1)	138 (80.2)	34 (19.8)	
	41–50	266 (26.4)	182 (68.4)	84 (31.6)	
	50 or more	223 (22.2)	132 (59.2)	91 (40.8)	
City of Residence					0.023
	Taif	463 (46)	331 (71.5)	132 (28.5)	
	Makkah	201 (20)	128 (63.7)	73 (36.3)	
	Jeddah	167 (16.6)	125 (74.9)	42 (25.1)	
	Madinah	134 (13.3)	90 (67.2)	44 (32.8)	
	Yanbu	41 (4.1)	35 (85.4)	6 (14.6)	
Educational Level					0.143
	Uneducated	11 (1.1)	7 (63.6)	4 (36.4)	
	Primary or intermediate school	31 (3.1)	21 (67.7)	10 (32.3)	
	High School	146 (14.5)	91 (62.3)	55 (37.7)	
	University student/bachelor’s degree	753 (74.9)	540 (71.7)	213 (28.3)	
	Higher Education	65 (6.5)	50 (76.9)	15 (23.1)	
Marital Status					0.422
	Single	317 (31.5)	232 (73.2)	85 (26.8)	
	Married	637 (63.3)	438 (68.8)	199 (31.2)	
	Divorced	39 (3.9)	30 (76.9)	9 (23.1)	
	Widow	13 (1.3)	9 (69.2)	4 (30.8)	
Employment Status					<0.001
	Unemployed	261 (25.9)	202 (77.4)	59 (22.6)	
	Student	160 (15.9)	113 (70.6)	47 (29.4)	
	Government employee	446 (44.3)	315 (70.6)	131 (29.4)	
	Retired	139 (13.8)	79 (56.8)	60 (43.2)	
Chronic Disease Status					0.079
	No chronic diseases	752 (74.8)	534 (71.1)	218 (28.9)	
	Have chronic diseases	254 (25.2)	175 (68.9)	79 (31.1)	
Monthly Income					0.171
	Less than 3000 SR	350 (34.8)	260 (74.3)	90 (25.7)	
	3000 to 6000 SR	148 (14.7)	103 (69.6)	45 (30.4)	
	7000 to 10,000 SR	123 (12.2)	89 (72.4)	34 (27.6)	
	More than 10,000 SR	385 (38.3)	257 (66.8)	128 (33.2)	
Lifestyle Factors					0.171
	Dietetic	103 (10.2)	82 (79.6)	21 (20.4)	
	Athletic	172 (17.1)	119 (69.2)	53 (30.8)	
	Smoker	67 (6.7)	43 (64.2)	24 (35.8)	
	Breastfeeding	21 (2.1)	16 (76.2)	5 (23.8)	
	Pregnant	14 (1.4)	12 (85.7)	2 (14.3)	

## Data Availability

The datasets used and analyzed during the current study are available from the corresponding author on reasonable request.

## References

[1] Elsahoryi N.A., Odeh M., Jadayil S.A., McGrattan A., Hammad F., Al-Maseimi O.D., Alzoubi K.H. (2023). Prevalence of dietary supplement use and knowledge, attitudes, practice (KAP) and associated factors in student population: A cross-sectional study. Heliyon.

[2] Alhomoud F., Mohammed B., Bondarev A.D. (2016). Knowledge, attitudes and practices (KAP) relating to dietary supplements among health sciences and non-health sciences students in one of the universities of United Arab Emirates (UAE). J. Clin. Diagn. Res..

[3] Alhazmi A., Kuriakose B.B., Mushfiq S., Muzammil K., Hawash M.M. (2023). Prevalence, attitudes, and practices of dietary supplements among middle-aged and older adults in Asir region, Saudi Arabia: A cross-sectional study. PLoS ONE.

[4] U.S. Food and Drug Administration *Dietary Supplement Labeling Guide: Chapter I. General Dietary Supplement Labeling*; U.S. Department of Health and Human Services: Silver Spring, MD, USA. https://www.fda.gov/food/dietary-supplements-guidance-documents-regulatory-information/dietary-supplement-labeling-guide-chapter-i-general-dietary-supplement-labeling.

[5] National Institutes of Health Office of Dietary Supplements *Dietary Supplement Health and Education Act of 1994 Public Law 103-417 103rd Congress*; U.S. Department of Health and Human Services: Bethesda, MD, USA. https://ods.od.nih.gov/About/DSHEA_Wording.aspx.

[6] Hassan S., Egbuna C., Tijjani H., Ifemeje J.C., Olisah M.C., Patrick-Iwuanyanwu K.C., Onyeike P.C., Ephraim-Emmanuel B.C. (2020). Dietary supplements: Types, health benefits, industry and regulation. Functional Foods and Nutraceuticals.

[7] Algaeed H.A., AlJaber M.I., Alwehaibi A.I., AlJaber L.I., Arafah A.M., Aloyayri M.A., Binsebayel O.A., Alotaiq S.A., Alfozan M.A., Ahmed I.B. (2019). General public knowledge and use of dietary supplements in Riyadh, Saudi Arabia. J. Fam. Med. Prim. Care.

[8] BlueWeave Consulting Saudi Arabia Dietary Supplements Market Size. https://www.blueweaveconsulting.com/report/saudi-arabia-dietary-supplements-market#:~:text=Saudi%20Arabia%20Dietary%20Supplements%20Market%20size%20was%20estimated%20at%20USD.

[9] El-Mouzan M., Al-Herbish A., Al-Salloum A., Foster P., Al-Omar A.A., Qurachi M.M., Kecojević T. (2010). Regional disparity in prevalence of malnutrition in Saudi children. Saudi Med. J..

[10] El Mouzan M.E., Foster P., Al Herbish A.A., Al Salloum A.A., Al Omar A.A., Qurachi M.M. (2010). Prevalence of malnutrition in Saudi children: A community-based study. Ann. Saudi Med..

[11] Alowais M.A., Selim M.A.E. (2019). Knowledge, attitude, and practices regarding dietary supplements in Saudi Arabia. J. Fam. Med. Prim. Care.

[12] Bailey R.L., Gahche J.J., Thomas P.R., Dwyer J.T. (2013). Why US children use dietary supplements. Pediatr. Res..

[13] National Institute on Aging *Dietary Supplements for Older Adults*; U.S. Department of Health and Human Services, Bethesda, MD, USA. https://www.nia.nih.gov/health/dietary-supplements-older-adults#50.

[14] Xiang C., Luo J., Yang G., Sun M., Liu H., Yang Q., Ouyang Y., Xi Y., Yong C., Khan M.J. (2022). Dietary supplement use during pregnancy: Perceptions versus reality. Int. J. Environ. Res. Public Health.

[15] National Institutes of Health Office of Dietary Supplements *Dietary Supplements for Exercise and Athletic Performance*; U.S. Department of Health and Human Services, Bethesda, MD, USA. https://ods.od.nih.gov/factsheets/ExerciseAndAthleticPerformance-HealthProfessional/.

[16] Kiani A.K., Dhuli K., Donato K., Aquilanti B., Velluti V., Matera G., Iaconelli A., Connelly S., Bellinato F., Gisondi P. (2022). Main nutritional deficiencies. J. Prev. Med. Hyg..

[17] Kourkouta L., Frantzana E., Iliadis C., Monios A., Dimitriadou A., Papathanassiou I.V. (2016). Health and dietary supplements. Int. J. Eng. Appl. Sci..

[18] National Institutes of Health Office of Dietary Supplements *Dietary Supplements: What You Need to Know*; U.S. Department of Health and Human Services, Bethesda, MD, USA. https://ods.od.nih.gov/factsheets/WYNTK-Consumer/#h3.

[19] Wooltorton E. (2003). Too much of a good thing? Toxic effects of vitamin and mineral supplements. CMAJ Can. Med. Assoc. J..

[20] AlTamimi J.Z. (2019). Awareness of the consumption of dietary supplements among students in a university in Saudi Arabia. J. Nutr. Metab..

[21] Quaidoo E., Ohemeng A., Amankwah-Poku M. (2018). Sources of nutrition information and level of nutrition knowledge among young adults in the Accra metropolis. BMC Public Health.

[22] Allehdan S., Hasan M., Perna S., Al-Mannai M., Alalwan T., Mohammed D., Almosawi M., Hoteit M., Tayyem R. (2023). Prevalence, knowledge, awareness, and attitudes towards dietary supplements among Bahraini adults: A cross-sectional study. Food Prod. Process. Nutr..

[23] Mohsen H., Yazbeck N., Al-Jawaldeh A., Bou Chahine N., Hamieh H., Mourad Y., Skaiki F., Salame H., Salameh P., Hoteit M. (2021). Knowledge, attitudes, and practices related to dietary supplementation, before and during the COVID-19 pandemic: Findings from a cross-sectional survey in the Lebanese population. Int. J. Environ. Res. Public Health.

[24] Terrie Y.C. (2019). Are women’s supplements and vitamins useful?. Pharm. Times.

[25] Ghazzawi H.A., Hazem E.S.A., Amawi A.T. (2022). Evaluation of knowledge, attitudes, and practices related to dietary supplements intake among college students: A cross-sectional study. J. Clin. Diagn. Res..

[26] Dickinson A., MacKay D. (2014). Health habits and other characteristics of dietary supplement users: A review. Nutr. J..

[27] Elder K., Nisly S. (2011). Dietary supplement education in a senior population. Open J. Intern. Med..

[28] Rawson E. (2005). Risks and benefits of supplement use. Curr. Sports Med. Rep..

[29] White C. (2020). Dietary supplements pose real dangers to patients. Ann. Pharmacother..

